# Supplementary material for: Comparison of Walking Quality Variables between End-Stage Osteonecrosis of Femoral Head Patients and Healthy Subjects by a Footscan Plantar Pressure System
Source: Medicina (Kaunas). 2022 Dec 28;59(1):59. doi: 10.3390/medicina59010059 (PMC9865786; doi:10.3390/medicina59010059)
Supplement: Supplementary file 1 [file medicina-59-00059-s001.zip › medicina-2041083-supplementary.pdf]

**Table S1** Static plantar pressure on the left and right side of the healthy group

| Variables | Left | Right | *p* Value |
|---|---|---|---|
| Forefoot (%) | 21.49±2.82 | 19.88±3.25 | 0.07 |
| Hindfoot (%) | 29.87±3.64 | 29.32±3.33 | 0.62 |
| Total (%) | 51.36±4.45 | 48.67±4.45 | 0.10 |

**Table S2** Dynamic plantar pressure distribution on the left and right side of the healthy group

| Variables | Left | Right | *p* Value |
|---|---|---|---|
| T1(N/cm$^2$) | 6.33±2.42 | 6.06±3.37 | 0.60 |
| T2-5(N/cm$^2$) | 2.37±1.80 | 2.13±1.52 | 0.28 |
| M1(N/cm$^2$) | 7.12±3.58 | 7.31±4.22 | 0.82 |
| M2(N/cm$^2$) | 13.28±3.76 | 13.35±4.05 | 0.91 |
| M3(N/cm$^2$) | 17.51±5.18 | 16.65±5.35 | 0.16 |
| M4(N/cm$^2$) | 11.60±4.40 | 10.70±4.97 | 0.07 |
| M5(N/cm$^2$) | 7.17±4.69 | 6.34±4.74 | 0.06 |
| MF(N/cm$^2$) | 4.53±1.73 | 4.03±1.50 | 0.06 |
| HM(N/cm$^2$) | 9.96±2.28 | 10.69±3.31 | 0.20 |
| HL(N/cm$^2$) | 10.22±3.22 | 9.58±2.85 | 0.26 |

**Table S3** Regional impulse percentage (%) on the left and right side of the healthy group

| Variables | Left | Right | *p* Value |
|---|---|---|---|
| T1(%) | 6.2±4.46 | 5.88±3.66 | 0.74 |
| T2-5(%) | 1.46±1.43 | 1.22±1.05 | 0.37 |
| M1(%) | 6.63±3.71 | 7.29±3.40 | 0.06 |
| M2(%) | 12.33±3.05 | 13.39±3.44 | 0.07 |
| M3(%) | 13.01±3.64 | 12.63±3.73 | 0.56 |
| M4(%) | 9.29±3.29 | 8.68±3.42 | 0.44 |
| M5(%) | 7.38±3.91 | 6.27±3.58 | 0.05 |
| MF(%) | 18.68±7.72 | 17.61±8.30 | 0.08 |
| HM(%) | 13.50±4.13 | 15.18±4.37 | 0.06 |
| HL(%) | 11.43±3.53 | 12.27±3.89 | 0.35 |

**Table S4** Regional contact area percentage (%) on the left and right side of the healthy group

| Variables | Left | Right | *p* Value |
|---|---|---|---|
| T1(%) | 10.53±1.76 | 9.90±2.28 | 0.28 |
| T2-5(%) | 7.24±3.29 | 7.24±3.29 | 0.67 |
| M1(%) | 7.54±1.22 | 8.31±2.61 | 0.15 |
| M2(%) | 6.93±0.51 | 7.42±1.49 | 0.09 |
| M3(%) | 5.65±0.44 | 5.98±1.06 | 0.10 |
| M4(%) | 5.69±0.51 | 5.92±0.95 | 0.26 |
| M5(%) | 7.54±1.13 | 7.11±1.56 | 0.20 |
| MF(%) | 28.12±2.87 | 27.03±3.97 | 0.29 |
| HM(%) | 11.26±1.27 | 11.66±1.35 | 0.32 |
| HL(%) | 9.50±1.14 | 9.93±0.99 | 0.16 |
